# Urethral Instability and Overactive Bladder Symptoms: Evidence From Clinical Practice

**DOI:** 10.7759/cureus.99207

**Published:** 2025-12-14

**Authors:** Sara D Freixo, Manuela Mira Coelho, Patrícia Pereira, João Sousa

**Affiliations:** 1 Department of Physical Medicine and Rehabilitation, Hospital de Braga, Braga, PRT

**Keywords:** female urethra, overactive bladder, urethral pressure, urge urinary incontinence, urodynamics criteria

## Abstract

Urethral instability (URI) is characterized by spontaneous fluctuations in resting urethral pressure, typically observed during the bladder filling phase in the absence of detrusor contractions. Despite decades of reports, URI remains under-recognized in clinical practice and absent from international guidelines. Some patients with refractory lower urinary tract symptoms (LUTS) may have underlying URI not identified by standard assessments focused on detrusor overactivity. This article presents three cases of patients with refractory LUTS, where urodynamic studies, including urethral profilometry, revealed urethral pressure instability associated with LUTS. The studies were conducted according to ICS (International Continence Society) standards. The findings support a potential pathophysiological role for the urethra in LUTS, independent of detrusor function. Recognition of URI may inform alternative therapeutic approaches, such as neuromodulation. Despite promising data, the lack of standardized definitions and methodologies hampers clinical implementation. URI may represent a distinct functional entity contributing to OAB symptoms. Incorporating urethral pressure assessment in urodynamics, especially in refractory cases, is essential to optimize diagnosis and treatment.

## Introduction

Urethral instability (URI) is a condition characterized by spontaneous and transient variations in resting urethral pressure that occur during the bladder filling phase, at rest, and in the absence of detrusor contractions [1,2]. Despite being reported more than four decades ago as a potentially relevant phenomenon in understanding urinary incontinence and urgency [3-5], URI remains a largely neglected condition in clinical practice and is rarely discussed in international guidelines [6,7].

Urethral pressure variations have been identified in several patients with lower urinary tract dysfunction (LUTD), including women with overactive bladder (OAB) syndrome, urge urinary incontinence (UUI), mixed urinary incontinence (MUI), and even stress urinary incontinence [5,8,9]. Population-based data indicate that roughly two-thirds of adults report at least one LUTS, that OAB affects about one in eight individuals with an age-related increase, and that urinary incontinence of any type is reported by approximately 25-45% of women [3,10]. This background prevalence underscores the clinical relevance of revisiting urethral mechanisms, such as URI, particularly in refractory presentations.

Although the traditional focus in the pathophysiology of urgency is on the detrusor muscle, several authors have shown that in some cases, the urethra may play a central role. Some studies suggest that a sudden drop in urethral pressure precedes the bladder contraction, indicating that urethral failure may act as an afferent trigger for the micturition reflex [4,11,12]. URI has also been shown to occur in the absence of detrusor contractions or signs of bladder overactivity [5,8,13]. These patients present with symptoms indistinguishable from those of OAB urgency, frequency, and urge incontinence, yet the underlying pathophysiology may involve local urethral mechanisms, such as reduced functional urethral length, decreased smooth muscle thickness and tone, or impaired central processing of urethral afferents, leading to abnormal sympathetic and/or parasympathetic reflex activation [7,14].

From a therapeutic perspective, the presence of URI may have significant implications. Studies on sacral neuromodulation have shown that patients with URI are more likely to respond positively compared to those without this urodynamic finding [14,15]. Furthermore, a reduction in URI has been observed after treatment with mirabegron, suggesting a possible indirect urethral modulation effect of β3-agonists [16].

Despite this evidence, urethral instability remains under-recognized in current clinical practice. The absence of standardized definitions and methodological consensus for its assessment limits its integration into diagnostic and therapeutic algorithms. The concept itself has historically been underestimated, partly due to difficulties in distinguishing physiological urethral pressure variations from those with clinical relevance [1,6].

In this context, the present article aims to present a series of illustrative urodynamic images demonstrating urethral instability in patients with refractory lower urinary tract symptoms (LUTS), such as urinary urgency and urge incontinence, in whom standard first-line treatments, including pelvic floor rehabilitation, antimuscarinic agents, and β3-adrenergic therapy, have failed. These images, obtained through urethral profilometry and documenting spontaneous urethral pressure variations, are intended to highlight the importance of recognizing urethral instability as a distinct functional diagnosis and to discuss its potential pathophysiological, diagnostic, and therapeutic implications in light of the existing literature.

## Case presentation

This article is based on the urodynamic observation of a series of patients with complaints of LUTS, evaluated in the context of LUTD. All included cases showed a URI finding in urodynamic studies, defined according to objective criteria described in the literature.

All cases presented clinical symptoms compatible with OAB syndrome, including urgency, urinary frequency, and urge incontinence; Complete urodynamic evaluation, including urethral pressure profilometry; presence of spontaneous variations ≥15 cmH2O in resting urethral pressure, observed during profilometry or pressure-flow studies with continuous urethral pressure measurement, consistent with urethral instability as previously described in the literature.

Urodynamic studies were performed using a triple-lumen catheter to record urethral pressure (UrP) and intravesical pressure, along with a rectal catheter to measure abdominal pressure, in accordance with the recommendations of the International Continence Society (ICS). Urethral profilometry was conducted with the patient in the sitting position, following the technique described by Brown and Wickham. The maximum resting urethral pressure curve was evaluated on profilometry, and continuous recording of UrP variations was carried out during the pressure-flow study, in cases 2 and 3, documenting their association with changes in detrusor pressure and the occurrence of urgency.

Case 1

**Figure 1:**
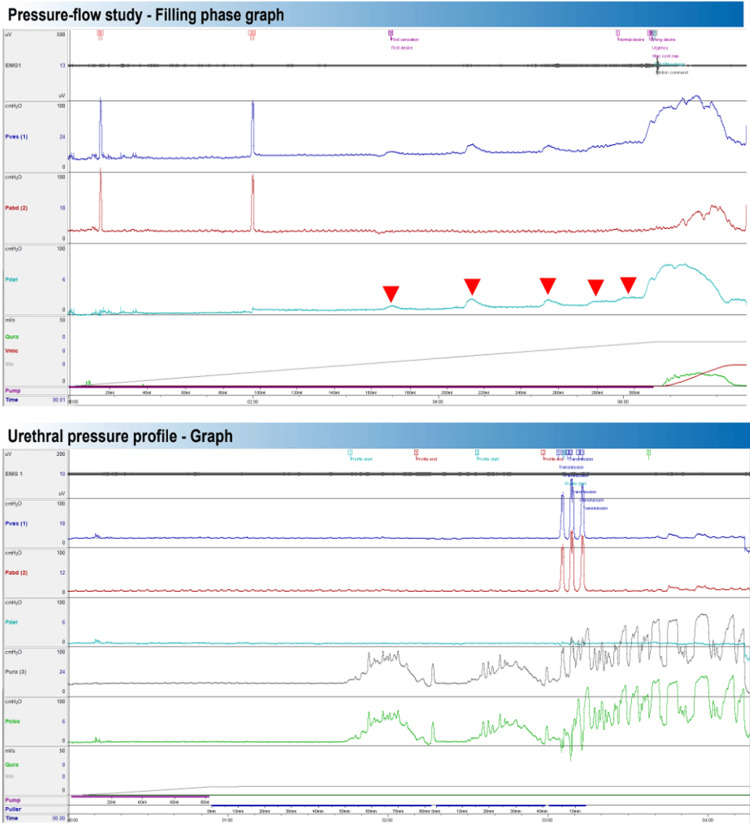
Case 1: Pressure-Flow and Urethral Pressure Profile Graph EMG: surface electromyography (pelvic floor activity);  Pves: vesical (intravesical) pressure; Pabd: abdominal (rectal) pressure; Pclos: urethral closure pressure; Pdet: detrusor pressure (Pdet = Pves − Pabd); Pura: urethral pressure; Vmic: voided volume

The patient was a 45-year-old woman with a past medical history including dyslipidemia, managed with statin therapy under the care of her general practitioner. Her obstetric history was one prior pregnancy with one live birth, delivered vaginally without complications. She presented with OAB symptoms, refractory to first-, second-, and third-line treatments, including intravesical botulinum toxin injection, with no improvement. Urodynamic evaluation revealed phasic detrusor overactivity during the pressure-flow study (red arrows). Urethral profilometry showed marked urethral instability, with urethral pressure variation exceeding 15 cmH₂O without detrusor contraction (Figure 1). Considering the patient’s symptoms, lack of response to previous treatments, and the urodynamic findings, she was referred for sacral nerve modulation. In this case, urethral pressure was not recorded during the pressure-flow phase, which could have provided additional relevant data for interpretation.

Case 2

**Figure 2:**
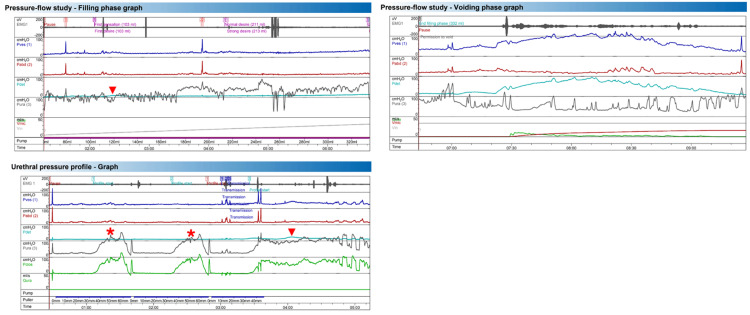
Case 2: Pressure-Flow and Urethral Pressure Profile Graph EMG: surface electromyography (pelvic floor activity);  Pves: vesical (intravesical) pressure; Pabd: abdominal (rectal) pressure; Pclos: urethral closure pressure; Pdet: detrusor pressure (Pdet = Pves − Pabd); Pura: urethral pressure; Vmic: voided volume

The patient was a 29-year-old woman with no prior pregnancies who presented with UUI, without voiding complaints or stress-related leakage, refractory to treatment with pelvic floor rehabilitation and trospium, with symptomatic improvement on mirabegron. During the pressure-flow study, spontaneous variations in urethral pressure were observed. At 120 mL of bladder filling, a phasic detrusor overactivity episode was recorded (red arrow), accompanied by a significant drop in urethral pressure (>15 cmH₂O) associated with the first sensation. The voiding phase showed an obstructive pattern with absent urethral sphincter relaxation, as evidenced by surface electromyography and urethral pressure assessment. Pressure-flow metrics supported functional outlet obstruction: Qmax 10.2 mL/s, Pdet.Qmax 48 cmH₂O, hesitancy 45 s, voiding time 103 s, average flow 3.0 mL/s, voided volume 311 mL, and computed residual 21 mL; maximum cystometric capacity was 332 mL. The urethral pressure profile revealed a polyphasic pattern on static profilometry (red asterisk), with detrusor overactivity (red arrow) occurring after a significant drop in urethral pressure (>15 cmH₂O) and failure to increase maximum urethral pressure during guarding effort, in the context of an overactive pelvic floor without relaxation, suggesting urethral instability (Figure 2).

Case 3

**Figure 3:**
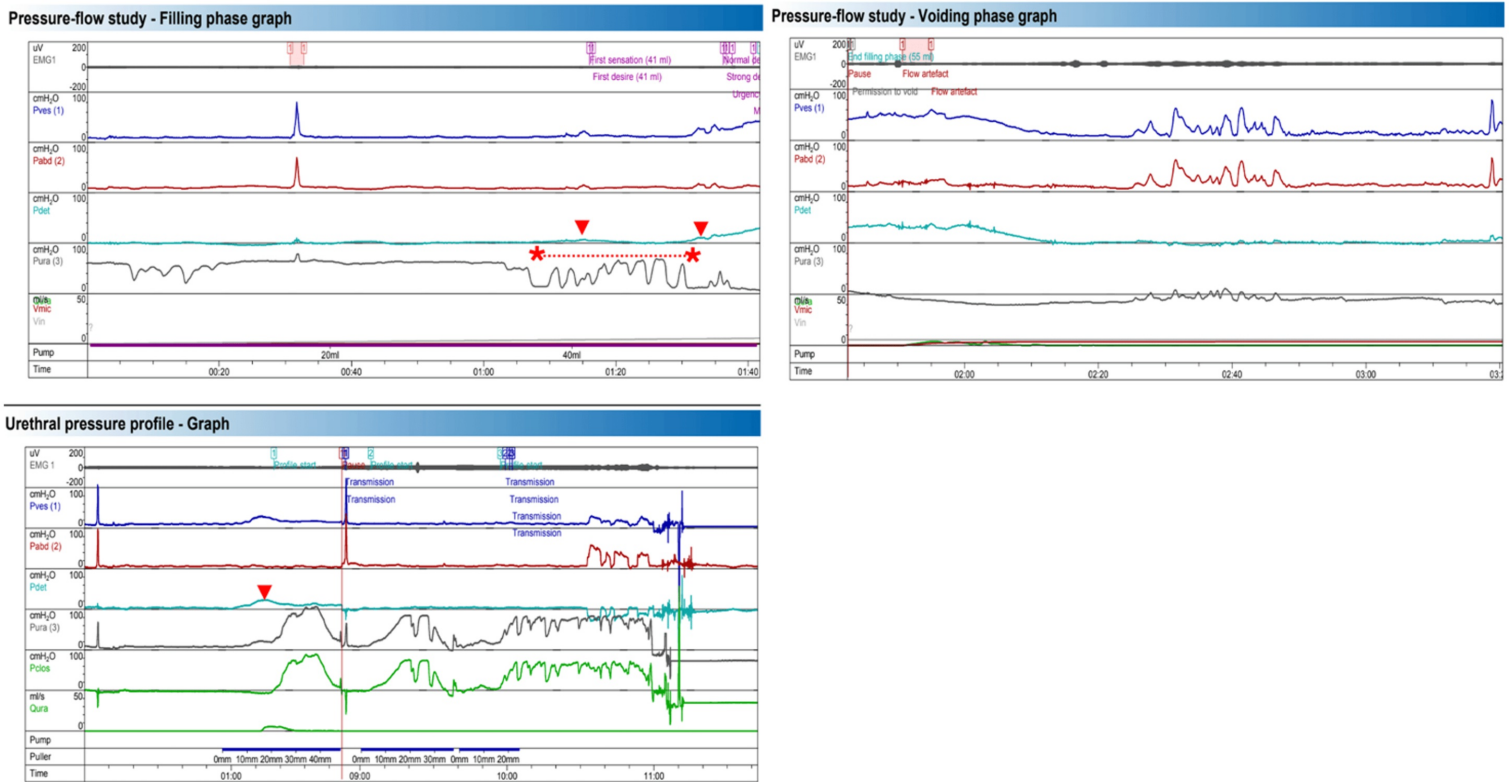
Case 3: Pressure-Flow and Urethral Pressure Profile Graph EMG: surface electromyography (pelvic floor activity);  Pves: vesical (intravesical) pressure; Pabd: abdominal (rectal) pressure; Pclos: urethral closure pressure; Pdet: detrusor pressure (Pdet = Pves − Pabd); Pura: urethral pressure; Vmic: voided volume

The patient was a 52-year-old woman with obesity; her current medication includes mirabegron. Her obstetric history was two prior pregnancies with two live births, both by cesarean section. She presented with UUI exclusively with storage symptoms, refractory to treatment with pelvic floor rehabilitation and antimuscarinic agents (trospium chloride and solifenacin), with only slight improvement on mirabegron. The pressure-flow study shows a drop in urethral pressure greater than 15 cmH₂O during the filling phase (highlighted between the red asterisks), followed by a detrusor contraction (red arrow) associated with the first sensation of urge at 35 mL. A second detrusor contraction occurred after another urethral pressure drop at 50 mL, accompanied by strong urgency and an uninhibited voiding episode. The static urethral pressure profile shows detrusor overactivity during initial catheter withdrawal and a polyphasic pressure pattern. The dynamic profile reveals marked urethral instability and a failure to increase pressure during the guarding effort. In this case, it remains unclear whether the sustained increase in urethral pressure observed during profilometry represents a compensatory mechanism to inhibit detrusor overactivity, an intrinsic sphincter deficiency, or primary urethral instability (Figure 3). This highlights the importance of interpreting urethral pressure variations in the context of comprehensive urodynamic findings and correlating them with the patient's clinical symptoms during evaluation.

## Discussion

URI was initially described by Petros and Ulmsten as a pattern of spontaneous variations in urethral pressure during the filling phase, in the absence of detrusor contractions, indicating a disturbance in the tonic function of the urethral sphincter [4]. Although the term is not formally incorporated into the ICS classifications, several authors have proposed objective diagnostic criteria, with the most accepted being the presence of sudden spontaneous variations ≥15 to 20 cmH2O in resting urethral pressure [5,8,9]. URI can be identified through urethral profilometry or on pressure-flow studies using continuous urethral pressure measurement, where such oscillations can be observed in patients with symptoms consistent with OAB, even in the absence of detrusor overactivity [9,13]. Despite methodological differences across studies, there is a growing consensus that these variations are pathological when exceeding 15-20 cmH2O, occur at rest, correlate with urgency and incontinence symptoms, and are associated with therapeutic failure [1,5,9]. Moreover, the term "urethral instability" has been used not only to describe manometric fluctuations but also a functional state of neuromuscular instability of the urethral sphincter, possibly related to disorganized sensory afferents or impaired central control of micturition [7,12]. Some large studies have shown that 7% to 11% of women with urgency and incontinence symptoms present with URI without evidence of involuntary detrusor contractions [5,8].

The pathophysiology of URI remains unclear, but several hypotheses have been proposed based on clinical studies, experimental models, and theoretical frameworks. One model by Petros and Ulmsten (1990) suggested that in some women with OAB symptoms, detrusor contraction is secondary to initial urethral failure, marked by a sudden drop in external sphincter pressure. This phenomenon, observed in urodynamic tracings, shows the role of the urethra acting as a potential afferent trigger for bladder activation [4,17]. The "guarding reflex failure" hypothesis gained support from observations that urethral pressure variations often precede bladder contractions in patients with urgency. The guarding reflex, mediated by tonic urethral afferents, inhibits detrusor activity during bladder filling and its dysfunction may remove this basal inhibition and allow uninhibited contractions to emerge. Another perspective suggests neurofunctional instability of the external urethral sphincter, related to sympathetic tone variations or central inhibition failure [12]. Sacral neuromodulation studies show that modulating urethral afferent input can significantly alter symptoms, suggesting that urethral-related neural circuits play a central role in pathophysiology [12,13].

URI is a condition whose identification depends on specific techniques for functional assessment of the urethra, as there are no direct structural or imaging markers. The gold standard remains resting urethral pressure profilometry, but measurement methodology directly affects results.

Despite the diagnostic value of urethral pressure variations, methodological inconsistencies remain a significant barrier to their widespread clinical use. This is largely due to the lack of standardization and reproducibility of urethral pressure profilometry, which is influenced by multiple variables such as catheter type, size and orientation, perfusion rate, withdrawal speed, patient position, bladder volume, and system response time. The International Consultation on Incontinence - Research Society (ICI-RS) in 2014 recommended that future studies aim to standardize technical parameters and establish normative values across different age groups and symptom profiles [7].

URI has been associated with LUTS, particularly prevalent in women with urgency, UUI, OAB, and MUI. Urethral profilometry-based studies have shown URI in up to 54% of women with LUTS and around 25% of those with idiopathic OAB [9,11].

Recognizing URI as a primary or contributing factor in urgency symptoms and UUI has important therapeutic implications. While most OAB treatments target detrusor control, available data suggest that urethra-focused interventions may be more effective in URI cases. Sacral neuromodulation has shown notable efficacy in these patients. In a prospective observational study, URI presence was a predictive marker of a favorable response to neuromodulation, with an 89% success rate versus 8% in patients without URI [14]. A follow-up study involving 100 patients confirmed that URI appeared to respond well to sacral neuromodulation; however, it was not identified as an independent predictor of treatment success [15]. The proposed mechanism involves inhibition of abnormal urethral afferent signals or restoration of the guarding reflex, stabilizing both the sphincter and bladder.

Response to IUU therapy is often unpredictable in patients with URI. Some individuals experience no improvement with pelvic floor rehabilitation and antimuscarinic agents, likely due to the drug's detrusor-focused action [3]. More recently, the effect of mirabegron (β3-agonist) on urethral urodynamic parameters was studied. After six weeks of treatment, significant reduction of urethral pressure variations was noted, suggesting a possible indirect sympathetic modulation effect on the urethra [16].

Despite decades of accumulated evidence, URI remains a controversial and under-recognized condition. It is absent from formal ICS classifications, not only due to the lack of standardized definitions and diagnostic criteria, with considerable methodological variability across studies, but also because its clinical interpretation remains unclear. It is still uncertain whether URI represents a primary or secondary pathophysiological factor of neuromuscular dysfunction, or simply a normal variant in some patients. Most existing studies are observational, with small samples and no control groups, limiting evidence strength. To date, no randomized controlled trials have compared URI-targeted interventions with standard OAB therapies. These gaps highlight the need for prospective, multicenter, methodologically robust studies with standardized measurement techniques and validated diagnostic criteria.

## Conclusions

In this series, we present illustrative urodynamic images from patients with refractory LUTS in whom the consistent finding was spontaneous urethral pressure variation on urethral profilometry and, in selected cases, during pressure-flow studies. Across cases, urethral pressure drops of ≥15-20 cmH₂O were temporally linked to urgency and, at times, preceded detrusor activity. These observations support the practical value of targeted urethral pressure assessment when routine evaluation is inconclusive. From an operational standpoint, clearer acquisition protocols and standardized reporting of urethral pressure patterns would facilitate interpretation and clinical communication. Future work should refine measurement techniques, define actionable thresholds and descriptors, and assess whether recognizing these patterns can improve diagnostic confidence and treatment selection.
